# *Robertkochia solimangrovi* sp. nov., isolated from mangrove soil, and emended description of the genus *Robertkochia*

**DOI:** 10.1099/ijsem.0.003970

**Published:** 2020-01-24

**Authors:** Ming Quan Lam, Maša Vodovnik, Maša Zorec, Sye Jinn Chen, Kian Mau Goh, Adibah Yahya, Madihah Md Salleh, Zaharah Ibrahim, Lili Tokiman, Simon J. McQueen-Mason, Neil C. Bruce, Chun Shiong Chong

**Affiliations:** ^1^​ Department of Biosciences, Faculty of Science, Universiti Teknologi Malaysia, 81310 Skudai, Johor, Malaysia; ^2^​ Biotechnical Faculty, University of Ljubljana, Groblje 3, 1230 Domzale, Slovenija; ^3^​ Johor National Parks Corporation, Kota Iskandar, 79575 Iskandar Puteri, Johor, Malaysia; ^4^​ Centre for Novel Agricultural Products, Department of Biology, University of York, Wentworth Way, York, YO10 5DD, UK

**Keywords:** *Robertkochia solimangrovi*, polyphasic taxonomy, *Flavobacteriaceae*, mangrove

## Abstract

To date, there is sparse information for the genus *Robertkochia* with *Robertkochia marina* CC-AMO-30D^T^ as the only described member. We report here a new species isolated from mangrove soil collected at Malaysia Tanjung Piai National Park and perform polyphasic characterization to determine its taxonomic position. Strain CL23^T^ is a Gram-negative, yellow-pigmented, strictly aerobic, catalase-positive and oxidase-positive bacterium. The optimal growth conditions were determined to be at pH 7.0, 30–37 °C and in 1–2 % (w/v) NaCl. The major respiratory quinone was menaquinone-6 (MK-6) and the highly abundant polar lipids were four unidentified lipids, a phosphatidylethanolamine and two unidentified aminolipids. The 16S rRNA gene similarity between strain CL23^T^ and *R. marina* CC-AMO-30D^T^ is 96.67 %. Strain CL23^T^ and *R. marina* CC-AMO-30D^T^ clustered together and were distinguished from taxa of closely related genera in 16S rRNA gene phylogenetic analysis. Genome sequencing revealed that strain CL23^T^ has a genome size of 4.4 Mbp and a G+C content of 40.72 mol%. Overall genome related indexes including digital DNA–DNA hybridization value and average nucleotide identity are 17.70 % and approximately 70%, below the cutoffs of 70 and 95%, respectively, indicated that strain CL23^T^ is a distinct species from *R. marina* CC-AMO-30D^T^. Collectively, based on the phenotypic, chemotaxonomic, phylogenetic and genomic evidences presented here, strain CL23^T^ is proposed to represent a new species with the name *Robertkochia solimangrovi* sp. nov. (KCTC 72252^T^=LMG 31418^T^). An emended description of the genus *Robertkochia* is also proposed.

*Flavobacteriaceae* is one of the widely spread bacterial families and is composed of 158 genera at the time of writing [1]. The genus *Robertkochia* was introduced by Hameed *et al*. in 2014 [2] as one of the new genera in the family *Flavobacteriaceae *. Until now, the genus consisted of a single species *Robertkochia marina* CC-AMO-30D^T^, which was isolated from surface seawater Collected at Taichung harbour, Taiwan [2]. The species was described as Gram-negative, strictly aerobic, orange-pigmented and with iso-C_15 : 0_, iso-C_15 : 1_ G and iso-C_17 : 0_ 3-OH as predominant fatty acids. The report for *Robertkochia* is scarce as the previous study only focused on taxonomic assignment with one species reported so far [2]. Furthermore, the genome of this genus and its prospective applications have not been studied or reported.

*Robertkochia* and many other members of the *Flavobacteriaceae* are halophilic or halotolerant bacteria that reside in diverse saline environments such as seawater, mangrove forest and marine sediment [3–5]. Mangroves are inter-tidal wetlands that connect terrestrial and marine ecosystems [6]. Due to periodic tidal flats, drastic changes in salinity and nutrient availability of the mangrove environment make it a unique ecosystem [7]. Free-living and symbiotic bacteria in such environments were found to play essential roles in maintaining mangrove ecosystem by, for example, recycling organic matter and biotransformation of minerals [8–10]. It was estimated that less than 5 % of species in mangrove environments have been described so far [11]. Therefore, it could be considered as an interesting area to be explored. In the present study, strain CL23^T^ was isolated from soil obtained from a mangrove forest located at Tanjung Piai National Park, Johor, Malaysia. This strain was characterized using a polyphasic approach (phenotypic, chemotaxonomic and genomic aspects) following the recommended guidelines [12, 13] and new criteria for classification [14] to elucidate its taxonomic position. The results indicated that strain CL23^T^ represents a new species within the genus *Robertkochia* and the name *Robertkochia solimangrovi* sp. nov. is proposed.

## Isolation and home habitat

Soil from the mangrove forest was sampled at Tanjung Piai National Park (GPS location: 1° 16′ 06.0″ N, 103° 30′ 31.2″ E) in September 2017 with permit (CJB F No. 734342) granted by Johor National Parks Corporation. The soil samples were serially diluted with sterile distilled water (10^−1^ to 10^−8^). The diluted sample (0.1 ml) was spread onto marine agar 2216 (MA; BD Difco) and incubated at 30–35 °C for 1–14 days. A yellow-pigmented strain designated as CL23^T^ was isolated from the MA and restreaked twice to obtain a pure culture. The strain was maintained in marine broth 2216 (MB; BD Difco) with 20 % (v/v) glycerol at –80 °C. Strain CL23^T^ was deposited at the Korean Collection for Type Cultures (KCTC) and the Belgian Co-ordinated Collections of Micro-organisms (BCCM) under accessions KCTC 72252^T^ and LMG 31418^T^, respectively. For comparative polyphasic taxonomy characterization, *R. marina* CC-AMO-30D^T^ (=JCM 18552^T^) was obtained from the Japan Collection of Microorganisms (JCM). Both strains were routinely cultured on MA and in MB at 30 °C for 48 h, unless specified otherwise.

## 16S rRNA gene phylogeny

Genomic DNA was extracted using the DNeasy Blood and Tissue kit (Qiagen) and purified by using the DNA Clean and Concentrator−25 (Zymo Research) by following the instruction manual. The 16S rRNA gene of strain CL23^T^ was amplified by PCR using universal primers: 27F (5′-AGAGTTTGATCMTGGCTCAG-′3) and 1525R (5′-AAGGAGGTGWTCCARCC-3′) [15]. The 16S rRNA gene was sequenced at Apical Scientific Pte. Ltd. (Seri Kembangan, Malaysia). After the sequencing, the raw sequences were trimmed, and then aligned using clustal_w. The nearly full-length 16S rRNA gene was searched against the EzBioCloud database for identification. The amplified 16S rRNA gene of strain CL23^T^ was also cross-checked with the genome data to ensure the acquisition of the full-length gene (1522 bp). The 16S rRNA gene of strain CL23^T^ (MK258111) had highest similarity (96.67%) to *R. marina* CC-AMO-30D^T^ (JX235674), which is below the accepted threshold of 98.7 % for species delineation [14]. The 16S rRNA gene similarity was less than 94 % between strain CL23^T^ and other members of closely related genera: *Joostella marina* En5^T^ (93.82%), *Joostella atrarenae* M1-2^T^ (93.82%), *Zhouia spongiae* HN-Y44^T^ (93.75%) and *Pustulibacterium marinum* E403^T^ (93.35%).

**Fig. 1.**
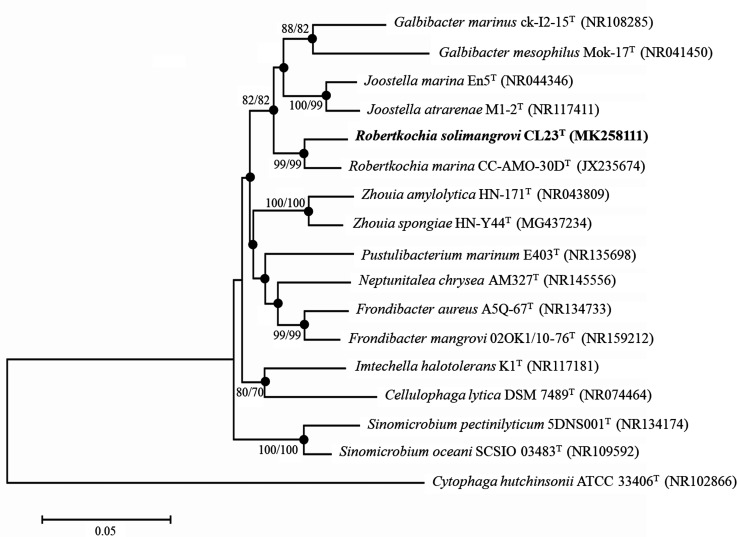
Neighbour-joining 16S rRNA gene phylogenetic tree manifesting the relationship of strain CL23^T^ with closely related taxa of the family *Flavobacteriaceae *. Corresponding GenBank accession numbers are indicated in parentheses. Bootstrap values ≥70 % based on 1000 resampled datasets are depicted as percentages at nodes. Bootstrap values are shown from left to right for NJ and ML results calculated with same sequence set. Filled circles indicate that corresponding nodes were also recovered in dendrograms generated using the ML algorithm. The sequence of *Cytophaga hutchinsonii* ATCC 33406^T^ was used as an outgroup. Bar, 0.05 substitutions per nucleotide position.

Phylogenetic trees of the 16S rRNA genes were reconstructed by using the neighbour-joining (NJ) [16] and maximum-likelihood (ML) [17] algorithms using mega 7.0 software [18] based on 1000 bootstrap replications [19] and Kimura's two-parameter model. The results of the 16S rRNA gene phylogenetic analysis (Fig. 1) demonstrated that strain CL23^T^ and *R. marina* CC-AMO-30D^T^ formed a clade in the NJ and ML trees, confirming the placement of strain CL23^T^ within the genus *Robertkochia *. The high bootstrap value at the node separating the branch containing strain CL23^T^ and *R. marina* CC-AMO-30D^T^ in 16S rRNA gene phylogenetic tree supported that these two strains are distinct from each other.

## Phenotypic and chemotaxonomic characterization

Colony morphology was observed on MA at 30 °C after 48 h incubation. Gram-staining was performed according to the protocol described previously [20]. Malachite green staining was used to assess the presence of endospore in 7 days old cultures [21]. Gram-stain reaction and endospore formation were examined under light microscope (Nikon Eclipse E200). Cell morphology was examined under scanning electron microscope (SEM; JSM-IT300LV, jeol). Bacterial motility was investigated by following the hanging-drop approach [22]. The presence of flexirubin-type pigment was determined by flooding the cells with 20 % (w/v) KOH [12].

Catalase activity was detected by effervescence using 3 % (v/v) H_2_O_2_ while oxidase activity was determined by oxidation of tetramethyl-*p*-phenylenediamine. Hydrolysis of starch, casein, ʟ-tyrosine, hypoxanthine, xanthine, Tween 20, Tween 40, Tween 60, Tween 80, carboxymethyl-cellulose (CMC) and xylan were tested according to Smibert and Krieg [21]. Bile aesculin hydrolysis was investigated using the method of Facklam and Moody [23]. Other biochemical characteristics were revealed by using API 20 E and API 20 NE kits (bioMérieux). Carbohydrate utilization and enzyme activity profiles of both strains were investigated by using API 50 CHB and API ZYM kits (bioMérieux), respectively. All API assays were carried out by following the manufacturer’s instructions with the slight modification that inoculation was supplemented up to 2 % (w/v) NaCl.

Growth under anaerobic conditions was tested by incubating the bacteria on MA for 14 days at 30 °C using AnaeroGen (Oxoid) in an anaerobic jar (Mitsubishi Gas Chemical). Growth was tested on the following media: Reasoner's 2A agar (R2A; HiMedia), nutrient agar (NA; Merck), tryptic soy agar (TSA; Merck), Luria–Bertani agar (LBA; Conda) and Mueller–Hinton agar (MHA; Sigma) supplemented with 2 % (w/v) NaCl at 30 °C for 7 days. The temperature range (4, 9, 15, 20, 25, 30, 37, 40, 42, 45 and 50 °C) and the optimum temperature for growth were determined using MB at pH 7. The pH range (in intervals of 1.0 pH unit) and optimum pH for growth were investigated using MB at 30 °C. The pH was adjusted with the following buffer systems: 50 mM citrate phosphate (pH 4–5), 50 mM sodium phosphate (pH 6–8) and 50 mM glycine–NaOH (pH 9–10) [24]. The pH was verified after autoclaving. To test NaCl tolerance and optimal concentration, the bacteria were grown in a medium containing yeast extract (1.0 g l^−1^), peptone (5.0 g l^−1^), MgCl_2_ (5.0 g l^−1^), MgSO_4_·7H_2_O (2.0 g l^−1^), CaCl_2_ (0.5 g l^−1^), KCl (1.0 g l^−1^) and NaCl (0, 0.5, 1–11 %, w/v) [10].

Antibiotic susceptibility of bacteria against 21 antibiotics was tested using the disc diffusion method on MA at 30 °C for 48 h [25]. The antibiotics discs (Oxoid) used were: ampicillin (10 µg), bacitracin (10 IU), carbenicillin (100 µg), chloramphenicol (100 µg), clindamycin (2 µg), doxycycline (30 µg), erythromycin (60 µg), gentamicin (10 µg), kanamycin (50 µg), lincomycin (2 µg), minocycline (30 µg), neomycin (30 µg), novobiocin (5 µg), oleandomycin (15 µg), oxacillin (1 µg), penicillin G (10 IU), piperacillin (100 µg), polymyxin B (300 IU), rifampicin (5 µg), streptomycin (10 µg) and tetracycline (30 µg).

Strain CL23^T^ was determined as a Gram-negative, rod-shaped, non-spore-forming, oxidase-positive and catalase-positive bacterium with motile ability by gliding. The colony was in a circular form with 0.5–1.0 mm diameter, a smooth surface, convex elevation, entire margin and had translucent property on MA after 48 h incubation. Under SEM, cells of strain CL23^T^ were 0.2–0.4 µm wide and 2.3–3.2 µm long. The notable distinctive features that differentiate strain CL23^T^ from *R. marina* CC-AMO-30D^T^ are shown in Table 1. In terms of morphology, strain CL23^T^ is yellow-pigmented while *R. marina* CC-AMO-30D^T^ was found to be orange-pigmented. Strain CL23^T^ grew well in 15–42 °C, pH 5–9 and 0–9 % (w/v) NaCl, and in general strain CL23^T^ demonstrated a broader growth range compared to *R. marina* CC-AMO-30D^T^ (Table 1). The optimal growth conditions of strain CL23^T^ were observed at 30–37 °C, pH 7 and 1–2 % (w/v) NaCl. Strain CL23^T^ was also able to produce acetoin, β-galactosidase and test results were weakly positive toward amygdalin according to the API 20 E assay, but not for *R. marina* CC-AMO-30D^T^. Based on API ZYM assay results, strain CL23^T^ was able to produce α-galactosidase, β-galactosidase and α-mannosidase, which were absent in *R. marina* CC-AMO-30D^T^. Both strains were further distinguished by the hydrolysis capability of gelatin, Tween 20, Tween 40 and Tween 60, and exhibiting resistance towards ampicillin, penicillin G, piperacillin and bacitracin (Table 1).

**Table 1.** Differential phenotypic characteristics of strain CL23^T^ and *Robertkochia marina* CC-AMO-30D^T^ Strains: 1, CL23^T^; 2, *R. marina* CC-AMO-30D^T^. All data were obtained from this study. +, Positive reaction; –, negative reaction; w, weakly positive reaction. All strains were positive for: catalase; hydrolysis of xylan and aesculin; production of acid from ᴅ-glucose, aesculin ferric citrate, cellobiose, maltose, sucrose, trehalose, melezitose, starch and glycogen in API 50 CHB strips; and activity of alkali phosphatase, esterase (C4), esterase lipase (C8), leucine arylamidase, valine arylamidase, cystine arylamidase, trypsin, chymotrypsin, acid phosphatase, naphtol-AS-BI-phosphohydrolase, α-glucosidase, β-glucosidase and *N*-acetyl-β-glucosaminidase. Both strains were negative for flexirubin-type pigment; growth under anaerobic condition; growth on R2A, NA, LBA, TSA and MHA media; hydrolysis of casein, starch, CMC, Tween 80, xanthine and hypoxanthine; nitrate reduction; indole and H_2_S production; urease; acid production from glycerol, erythritol, ᴅ-arabinose, ʟ-arabinose, ᴅ-ribose, ᴅ-xylose, ʟ-xylose, ᴅ-adonitol, methyl β-ᴅ-xylopyranoside, ᴅ-fructose, ʟ-sorbose, ʟ-rhamnose, dulcitol, inositol, ᴅ-mannitol, ᴅ-sorbitol, methyl α-ᴅ-mannopyranoside, *N*-acetyl-glucosamine, amygdalin, inulin, xylitol, ᴅ-lyxose, ᴅ-tagatose, ᴅ-fucose, ʟ-fucose, ᴅ-arabitol, ʟ-arabitol, potassium gluconate, potassium 2-ketogluconate and potassium 5-ketogluconate in API 50 CHB strips; and activity of lipase (C14) and β-glucuronidase (API ZYM).

Characteristics	1	2
Colony pigmentation	Yellow	Orange
Oxidase activity	+	–
Growth parameters:		
pH range	5–9	6–7
Temperature range (°C)	15–42	20–40
Temperature optimum (°C)	30–37	30
NaCl range (%, w/v)	0–9	0.5–4
NaCl optimum (%, w/v)	1–2	2
Hydrolysis of:		
Tween 20	+	w
Tween 40	+	–
Tween 60	+	w
Tyrosine	w	–
Gelatin	–	+
Production of acetoin	+	–
Oxidation of amygdalin	w	–
Utilization of:		
ᴅ-Galactose	+	–
ᴅ-Mannose	+	–
Arbutin	w	–
Salicin	w	–
Lactose	+	–
Melibiose	+	–
Raffinose	+	–
Gentiobiose	+	–
ᴅ-Turanose	w	–
Enzyme activity (API ZYM):		
α-Galactosidase	+	–
β-Galactosidase	+	–
α-Mannosidase	+	w
α-Fucosidase	w	–
Antibiotic susceptibility (per disc):		
Ampicillin (10 µg)	–	+
Penicillin G (10 IU)	–	+
Piperacillin (100 µg)	–	+
Bacitracin (45 µg)	–	+

For the chemotaxonomic analysis, cellular fatty acids were extracted following the protocol of Microbial Identification System (midi, version 6.1) [26]. Biomass of strain CL23^T^ and its reference strain *R. marina* CC-AMO-30D^T^ were harvested from MA after 48 h of incubation at 30 °C. The cells were saponified with a methanolic base, then the resulting sodium salts of fatty acids were methylated. In the final step, methyl esters were transferred to the organic phase and washed. Fatty acid methyl esters were analysed on Agilent 6890 apparatus equipped with an Ultra-2 capillary column and subsequently identified in the RTSBA6 library. As exhibited in Table 2, the predominant cellular fatty acids of strain CL23^T^ and *R. marina* CC-AMO-30D^T^ were found to be iso-C_15 : 0_, iso-C_15 : 1_ G and iso-C_17 : 0_ 3-OH (>10 %). Nonetheless, some fatty acid patterns and abundance of strain CL23^T^ varied when compared to *R. marina* CC-AMO-30D^T^, such as summed features 3 (3.64 %) and 9 (5.24 %) were detected in strain CL23^T^ but none for *R. marina* CC-AMO-30D^T^. In addition, the amounts of iso-C_16 : 0_, anteiso-C_15 : 0_ and iso-C_16 : 0_ 3-OH in strain CL23^T^ were remarkably lower than those in *R. marina* CC-AMO-30D^T^ (Table 2).

**Table 2.** Cellular fatty acid content (%) of strain CL23^T^ and *Robertkochia marina* CC-AMO-30D^T^ Strains: 1, CL23^T^; 2, *R. marina* CC-AMO-30D^T^. All data presented in the table are from this study. tr, Trace (≤0.5 %); –, not detected. Major components (>10 %) are highlighted in bold.

Fatty acid	1	2
Branched saturated:		
iso-C_13 : 0_	tr	2.4
iso-C_14 : 0_	–	2.4
iso-C_15 : 0_	**21.8**	**19.9**
iso-C_16 : 0_	3.4	6.1
anteiso-C_15 : 0_	2.3	5.8
Unsaturated:		
C_15 : 1_ω5*c*	0.7	–
C_17 : 1_ω6*c*	1.7	–
C_17 : 1_ω8*c*	0.8	–
Branched unsaturated:		
iso-C_15 : 1_ G	**10.8**	**23.3**
iso-C_16 : 1_ G	–	1.6
iso-C_16 : 1_ h	1.0	–
anteiso-C_15 : 1_ A	tr	2.8
Hydroxy:		
C_15 : 0_ 2-OH	0.9	1.5
C_15 : 0_ 3-OH	2.0	0.6
C_16 : 0_ 3-OH	1.4	tr
C_17 : 0_ 3-OH	1.1	tr
iso-C_16 : 0_ 3-OH	2.6	6.5
iso-C_17 : 0_ 3-OH	**29.5**	**15.5**
Summed features:*		
3†	3.6	–
9‡	5.2	–

*Summed features are groups of two or three fatty acids that cannot be separated by GLC with the midi system.

†Summed feature 3 consisted of iso-C_15 : 0_ 2-OH, C_16 : 1_ω6*c* and/or C_16 : 1_ω7*c* and annotated here as iso-C_15 : 0_ 2-OH based on the equivalent chain length (ECL).

‡Summed feature 9 consisted of iso-C_17 : 1_ ω9*c* and/or C_16 : 0_ 10-methyl.

The polar lipids and respiratory quinone analyses of strain CL23^T^ were performed by Dr. Brian Tindall at the Identification Service, DSMZ, Braunschweig, Germany. In brief, the respiratory quinones were extracted by solvent methanol : hexane (2 : 1 v/v), separated by TLC and HPLC following the standard method by Tindall [27]. The polar lipids were extracted using chloroform : methanol solvent and separated by two-dimensional silica gel TLC [28]. Total lipid material was identified using molybdatophosphoric acid and specific functional groups were determined using spray reagents specific for defined functional groups.

The major respiratory quinone of strain CL23^T^ was identified to be menaquinone-6 (MK-6), which matched *R. marina* [2] and other members in the family *Flavobacteriaceae* [12]. In terms of polar lipids, strain CL23^T^ had four unidentified lipids (L1, L2, L3 and L4), a phosphatidylethanolamine and two unidentified aminolipids (AL1 and AL2) as major polar lipids (Fig. S1, available in the online version of this article). Additionally, three unidentified glycolipids (GL1, GL2 and GL3) and an unknown lipid (L5) were observed in minor amounts. The unidentified lipids (L1–L3) and glycolipids (GL1–GL3) were not detected in *R. marina* CC-AMO-30D^T^ [2]. Moreover, an unidentified phospholipid was detected in *R. marina* CC-AMO-30D^T^, which was not found in strain CL23^T^ [2].

## Genomic characterization

The genome of reference strain *R. marina* CC-AMO-30D^T^ was not available at the time of study, therefore, both the genomes of strain CL23^T^ (NCBI accession: QKWN00000000) and *R. marina* CC-AMO-30D^T^ (NCBI accession: QXMP00000000) were sequenced in this study. Whole genome sequencing of strain CL23^T^ was accomplished on an Illumina HiSeq 2500 platform (2×150 bp). The raw reads were filtered, and the quality data was *de novo* assembled using SOAPdenovo 2.04 [29]. The resulting genome was annotated using the ncbi Prokaryotic Genome Annotation Pipeline (PGAP) [30].

The assembled genome of strain CL23^T^, consisting of 23 contigs with 322× depth of sequencing coverage (average), made up the size of genome with 4 407 290 bp and a G+C content of 40.72 mol%. The genome size of strain CL23^T^ is significantly larger than that of *R. marina* CC-AMO-30D^T^ (3 571 649 bp). The G+C content of strain CL23^T^ is slightly lower than that of *R. marina* CC-AMO-30D^T^ (43.67 mol%). Based on pgap annotation, a total of 3669 protein-coding genes was found in the genome of strain CL23^T^. The genes responsible for phosphatase activity were found in the genome of strain CL23^T^ and *R. marina* CC-AMO-30D^T^ with a total of 12 and 7 phosphatases encoded, respectively (Table S1). This correlated to the API ZYM results in which both strains were positive to acidic and alkali phosphatases. Notably, the number of phosphatases annotated is higher in strain CL23^T^ as compared to *R. marina* CC-AMO-30D^T^. On the other hand, strain CL23^T^ consists of a series of genes for assimilatory sulfate reduction into sulfite (sulfate adenylyltransferase subunit CysN and CysD, adenylylsulfate kinase and phosphoadenylylsulfate reductase) and then sulfite reduction into sulfide (FAD-binding oxidoreductase and LLM class flavin-dependent oxidoreductase) (Table S1). Nevertheless, the genes responsible for reduction of sulfite to sulfide are absent in *R. marina* CC-AMO-30D^T^ (Table S1). Furthermore, strain CL23^T^ also encodes a set of genes for reduction of nitrate to ammonia (*NirBD* and *NrfAH*) in which *NirBD* genes were not found in genome of *R. marina* CC-AMO-30D^T^ (Table S1). These genes suggest that strain CL23^T^ participates in nutrient recycling in mangrove environments.

**Fig. 2.**
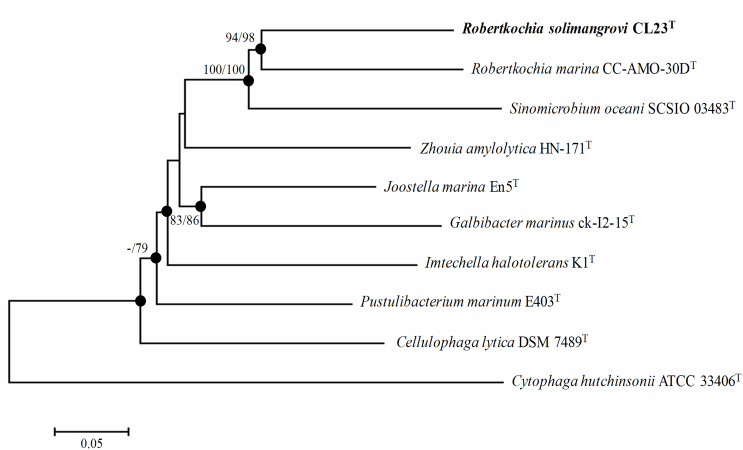
Neighbour-joining phylogenetic tree based on the concatenated sequences of five housekeeping genes: *rpoB–gyrB–recA–mutL–atpD*, indicating the position of strain CL23^T^. Bootstrap values ≥70 % based on 1000 resampled datasets are depicted as percentages at nodes; value <70 % is indicated by a dash. Bootstrap values from left to right for the NJ and ML results calculated with the same sequence set. Filled circles indicate that the corresponding nodes were also recovered in dendrograms generated using the ML algorithm. The sequence of *Cytophaga hutchinsonii* ATCC 33406^T^ was used as an outgroup. Bar, 0.05 substitutions per nucleotide position.

Multilocus sequence analysis (MLSA) was conducted on five housekeeping genes of strain CL23^T^, *R. marina* CC-AMO-30D^T^ and related genera, which the sequences were retrieved from genome data. The sequences of housekeeping genes were aligned individually and then concatenated in the following order: *rpoB–gyrB–recA–mutL–atpD*. The phylogenetic tree of concatenated housekeeping genes was reconstructed using mega 7.0 similarly as described above. In this tree (Fig. 2), strain CL23^T^ and *R. marina* CC-AMO-30D^T^ are clustered together but well distinguished from each other with high level of support (>90 % bootstrap value). Likewise, the phylogenetic tree based on whole genome sequences that was built using realphy 1.12 [31] also supported the finding that both strain CL23^T^ and *R. marina* CC-AMO-30D^T^ are grouped within the same clade (Fig. S2).

To further underpin the classification of strain CL23^T^ as representing a new species, the overall genome related indexes (OGRIs) were determined. Average nucleotide identity based on blast (ANIb) was calculated using JSpeciesWS [32]. ANI based on usearch (OrthoANIu) was determined by using ChunLab's online ANI calculator [33]. The digital DNA–DNA hybridization (dDDH) value was calculated by using the Genome-to-Genome Distance Calculator [34].

The ANIb and OrthoANIu values between strain CL23^T^ and *R. marina* CC-AMO-30D^T^ were 69.35 and 70.47% respectively. These ANI values are below the recommended threshold of 95–96 % for species delineation [35]. Similarly, the dDDH value between two strains was found to be 17.70%, lower than 70%, the cut-off for species boundaries [34]. Combining the interpretation of ANI and dDDH values, the result revealed the identity of strain CL23^T^ as a distinct species within the same genus as *R. marina* CC-AMO-30D^T^.

Based on polyphasic taxonomy characterization including phenotypic, chemotaxonomic, phylogenetic and genomic aspects, the results clearly indicated that strain CL23^T^ (=KCTC 72252^T^=LMG 31418^T^) represents a new species within the genus *Robertkochia *, for which the name *Robertkochia solimangrovi* sp. nov. is proposed.

## Description of *Robertkochia solimangrovi* sp. nov.

*Robertkochia solimangrovi* sp. nov. (so.li.man.gro′vi. L. neut. n. *solum* soil; N.L. neut. n. *mangrovum* a mangrove; N.L. gen. n. *solimangrovi* of soil of a mangrove, pertaining to where the type strain was isolated).

The cells are Gram-negative, rod-shaped, approximately 0.2–0.4 µm wide, 2.3–3.2 µm long and motile by gliding. Colonies are yellow-pigmented, circular, 0.5–1.0 mm in diameter, smooth, convex, have an entire margin and translucent after 48 h incubation at 30 °C on MA. Flexirubin-type pigment is absent. Cells are positive for oxidase and catalase. Growth occurs at 15–42 °C (optimum, 30–37 °C), pH 5–9 (optimum, pH 7) and in the presence of 0–9 % (w/v) NaCl [optimum, 1–2 % (w/v) NaCl]. Grows well on MA, however, no growth is observed on R2A, NA, LBA, TSA and MHA media. No growth is observed on MA under the anaerobic condition. The predominant fatty acids are iso-C_15 : 0_, iso-C_15 : 1_ G and iso-C_17 : 0_ 3-OH. The major isoprenoid quinone is menaquinone-6 (MK-6). The major polar lipids are four unidentified lipids, a phosphatidylethanolamine and two unidentified aminolipids. Xylan, aesculin, Tween 20, Tween 40 and Tween 60 are hydrolysed. ʟ-Tyrosine is weakly hydrolysed. Casein, starch, CMC, Tween 80, xanthine and hypoxanthine are not hydrolysed. In the API 20 E strip, positive for ΟNP-β-ᴅ-galactopyranoside and acetoin production; weakly positive for fermentation/oxidation of amygdalin; negative for arginine dihydrolase, lysine decarboxylase, ornithine decarboxylase, tryptophane deaminase, urease and gelatinase, production of H_2_S and indole, utilization of citrate, fermentation/oxidation of glucose, mannitol, inositol, sorbitol, rhamnose, sucrose, melibiose and ʟ-arabinose. In the API 20 NE strip, positive for hydrolysis of pNP-β-ᴅ-galactopyranoside and aesculin ferric citrate; negative for nitrate reduction, indole production, arginine dihydrolase, gelatinase and urease, fermentation of glucose and assimilation of glucose, arabinose, mannose, ᴅ-mannitol, *N*-acetyl-glucosamine, maltose, potassium gluconate, capric acid, adipic acid, malic acid and phenylacetic acid. In the API 50 CHB strip, acid is produced from galactose, glucose, mannose, aesculin ferric citrate, cellobiose, maltose, lactose, melibiose, sucrose, trehalose, melezitose, raffinose, starch, glycogen and gentibiose; acid is weakly produced from methyl α-glucopyranoside, arbutin, salicin and turanose; acid is not produced from glycerol, erythritol, arabinose, ʟ-arabinose, ribose, xylose, xylose, adonitol, methyl β-xylopyranoside, fructose, ʟ-sorbose, ʟ-rhamnose, dulcitol, inositol, mannitol, sorbitol, methyl α-mannopyranoside, *Ν*-acetyl-glucosamine, amygdalin, inulin, xylitol, lyxose, tagatose, fucose, fucose, arabitol, arabitol, potassium gluconate, potassium 2-ketogluconate and potassium 5-ketogluconate. In the API ZYM strip, alkali phosphatase, esterase (C4), esterase lipase (C8), leucine arylamidase, valine arylamidase, cystine arylamidase, trypsin, chymotrypsin, acid phosphatase, naphtol-AS-BI-phosphohydrolase, α-galactosidase, β-galactosidase, α-glucosidase, β-glucosidase, *N*-acetyl-β-glucosaminidase and α-mannosidase are present; weak positive reaction for α-fucosidase and negative results for lipase (C14) and α-fucosidase. Cells are susceptible to carbenicillin, clindamycin, doxycycline, lincomycin, minocycline, novobiocin, oleandomycin, rifampicin and tetracycline, but not to ampicillin, bacitracin, chloramphenicol, erythromycin, gentamicin, kanamycin, neomycin, oxacillin, penicillin G, piperacillin, polymyxin B and streptomycin.

The type strain is CL23^T^ (=KCTC 72252^T^=LMG 31418^T^), isolated from soil of mangrove collected from Tanjung Piai National Park, Johor, Malaysia. Genome metrics are as follows: genome size, 4 407 290 bp; number of contigs, 23; G+C content, 40.72 mol%.

## Emended description of the genus *Robertkochia* Hameed *et al*. 2014

The characteristics of the genus *Robertkochia* are described according to Hameed *et al*. 2014 [2] with following amendments and additional information. Oxidase is either positive or negative and catalase is positive. The DNA G+C content of the type strain of type species is 43.67 mol% based on genome data. The Whole Genome Shotgun project of type strain of type species is available at EMBL/DDBJ/GenBank under accession QXMP00000000. The version described in this paper is QXMP01000000.

## Supplementary Data

Supplementary material 1
