# Supplementary material for: Robertkochia solimangrovi sp. nov., isolated from mangrove soil, and emended description of the genus Robertkochia
Source: Int J Syst Evol Microbiol. 2020 Jan 24;70(3):1769–76. doi: 10.1099/ijsem.0.003970 (PMC7386787; doi:10.1099/ijsem.0.003970)
Supplement: Supplementary material 1 [file ijsem-70-1769-s001.pdf]

Supplementary Materials

Robertkochia solimangrovi **sp. nov., isolated from mangrove soil, and emended description of the genus *Robertkochia***

Ming Quan Lam[1], Maša Vodovnik[2], Maša Zorec[2], Sye Jinn Chen[1], Kian Mau Goh[1], Adibah Yahya[1], Madihah Md Salleh[1], Zaharah Ibrahim[1], Lili Tokiman[3], Simon J. McQueen-Mason[4], Neil C. Bruce[4*] and Chun Shiong Chong[1*]

[1] Department of Biosciences, Faculty of Science, Universiti Teknologi Malaysia, 81310 Skudai, Johor, Malaysia

[2] Biotechnical Faculty, University of Ljubljana, Groblje 3, 1230 Domzale, Slovenija

[3] Johor National Parks Corporation, Kota Iskandar, 79575 Iskandar Puteri, Johor, Malaysia

[4] Centre for Novel Agricultural Products, Department of Biology, University of York, Wentworth Way, York, YO10 5DD, United Kingdom

***Correspondence:** Chun Shiong Chong, cschong@utm.my; Neil C. Bruce, neil.bruce@york.ac.uk

Supplementary figures

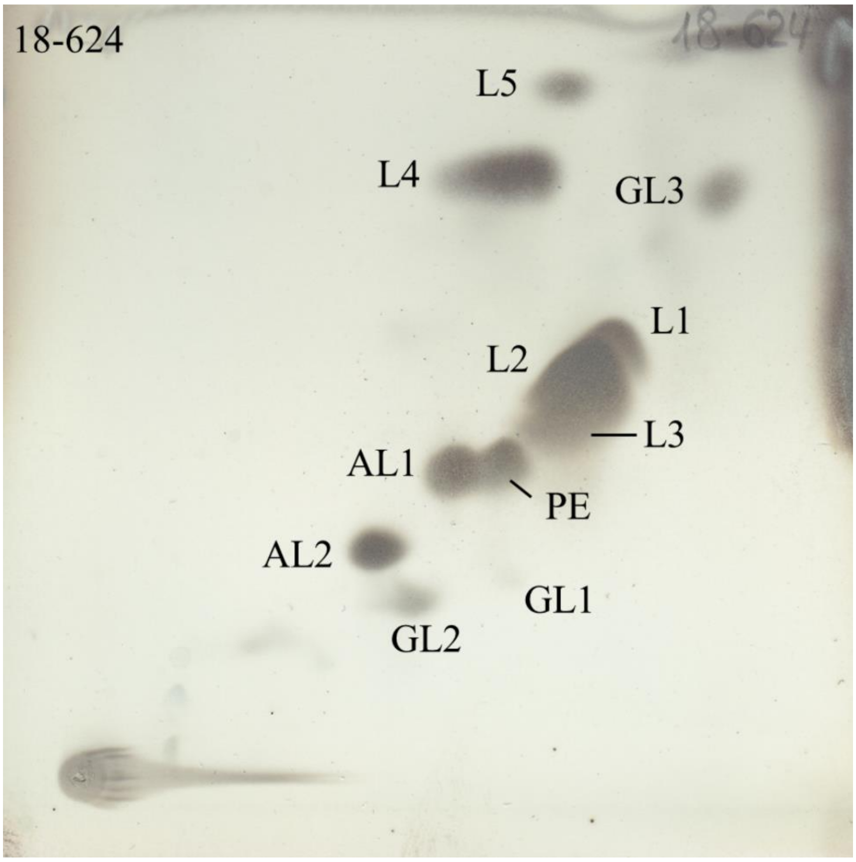

Fig. S1. Polar lipids profile of strain CL23^{T}. Unidentified lipids; L1–L5, phosphatidylethanolamine; PE, unidentified aminolipids; AL1–AL2, unidentified glycolipids; GL1–GL3.

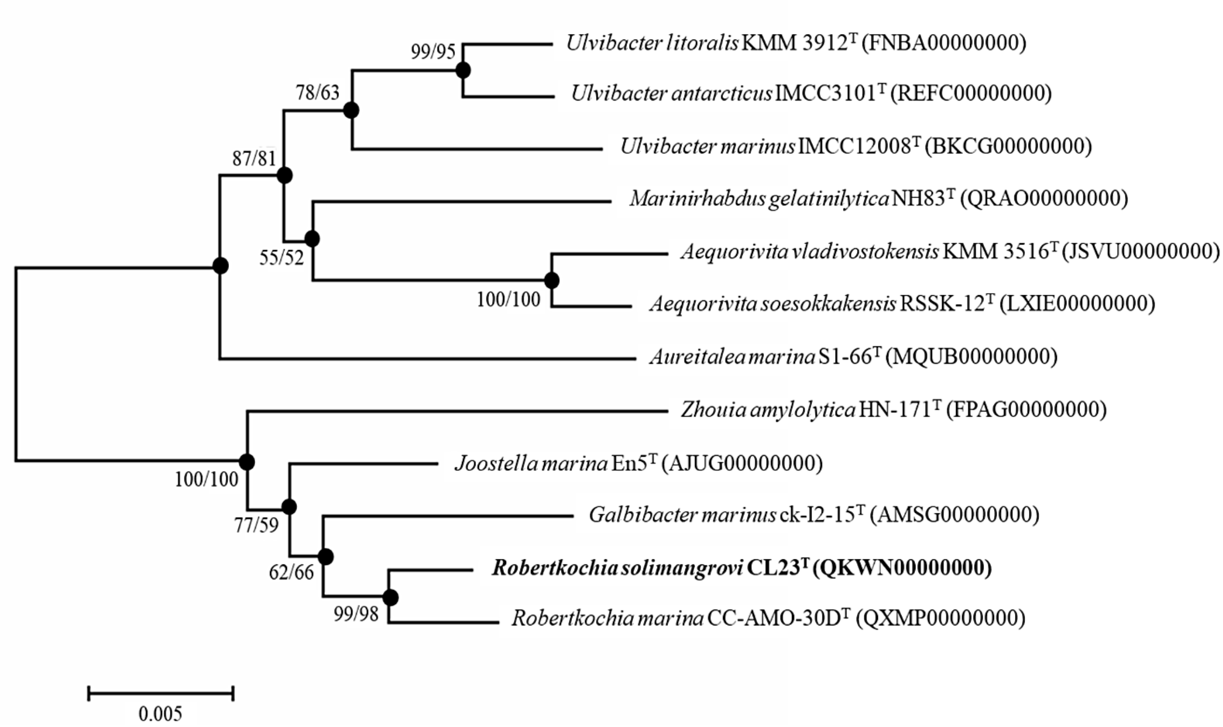

Fig. S2. Neighbor joining phylogenomic tree manifesting the relationship of strain CL23^T with closely related taxa of family *Flavobacteriaceae*. Corresponding Genbank accession numbers are indicated in parentheses. Bootstrap values ≥50% based on 1000 resampled datasets are depicted as percentages at nodes. Bootstrap value from left to right for NJ and ML calculated with same sequence set. Filled circles indicate that corresponding nodes were also recovered in dendrograms generated using ML algorithm. Bar, 0.005 substitutions per nucleotide position.

Supplementary tables

Table S1. List of potential genes for phosphatases, sulfur reduction and nitrate reduction encoded in the genome of strain CL23^T and *R. marina* CC-AMO-30D^T.

Category	Bacterial strain	NCBI Annotation	Accession
Phosphatases	CL23^T	alkaline phosphatase family protein	TRZ44267
		alkaline phosphatase	TRZ44500
		alkaline phosphatase	TRZ44343
		pyrophosphatase	TRZ44378
		sodium-translocating pyrophosphatase	TRZ44400
		alkaline phosphatase family protein	TRZ43533
		alkaline phosphatase family protein	TRZ43596
		alkaline phosphatase	TRZ42861
		HAD family phosphatase	TRZ42760
		alkaline phosphatase family protein	TRZ42969
		alkaline phosphatase family protein	TRZ41972
		HAD family phosphatase	TRZ41063
	R. marina CC-AMO-30D^T	alkaline phosphatase family protein	TRZ46762
		alkaline phosphatase family protein	TRZ45488
		alkaline phosphatase family protein	TRZ45685
		sodium-translocating pyrophosphatase	TRZ44743
		HAD family phosphatase	TRZ42656
		pyrophosphatase	TRZ41149
		HAD family phosphatase	TRZ40862
Sulfur reduction	CL23^T	sulfate adenylyltransferase subunit CysN	TRZ46029
		sulfate adenylyltransferase subunit CysD	TRZ46030
		adenylyl-sulfate kinase	TRZ46031
		phosphoadenylylsulfate reductase	TRZ44200
		phosphoadenylylsulfate reductase	TRZ42776
		FAD-binding oxidoreductase	TRZ41175
		LLM class flavin-dependent oxidoreductase	TRZ41182
	R. marina CC-AMO-30D^T	sulfate adenylyltransferase subunit CysN	TRZ40960
		sulfate adenylyltransferase subunit CysD	TRZ40970
		adenylyl-sulfate kinase	TRZ40959
		phosphoadenylylsulfate reductase	TRZ46694
Nitrate reduction	CL23 ^{T}	nitrite reductase (*NirBD*)	TRZ44395
		nitrite reductase (NAD(P)H) (*NirBD*)	TRZ42280

	nitrite reductase (NAD(P)H) small subunit (*NirBD*)	TRZ42281
	NAD(P)H-nitrite reductase (*NirBD*)	TRZ42287
	ammonia-forming cytochrome c nitrite reductase (*NrfAH*)	TRZ42033
	cytochrome c nitrite reductase small subunit (*NrfAH*)	TRZ42034
R. marina CC-AMO-30D^T	ammonia-forming cytochrome c nitrite reductase (*NrfAH*)	TRZ44150
	cytochrome c nitrite reductase small subunit (*NrfAH*)	TRZ44178
